# Siddha Medicine in Eastern Sri Lanka Today–Continuity and Change in the Treatment of Diabetes

**DOI:** 10.3389/fphar.2018.01022

**Published:** 2018-10-10

**Authors:** Saravanan V. Sathasivampillai, Pholtan R. S. Rajamanoharan, Michael Heinrich

**Affiliations:** ^1^Pharmacognosy and Phytotherapy, UCL School of Pharmacy, London, United Kingdom; ^2^Planning Unit, Provincial Department of Indigenous Medicine, Trincomalee, Sri Lanka

**Keywords:** Sri Lanka, diabetes mellitus, Tamil medicine, *Syzygium cumini*, Fabaceae, Eastern Province, ethnobotany, Siddha Medicine

## Abstract

Diabetes is affecting the social and economic developments in developing countries like Sri Lanka. Siddha Medicine (Tamil Medicine) is mostly practiced in the Eastern and Northern Provinces of Sri Lanka. Our recent review of Sri Lankan Siddha historical documents identified 171 plant species used to prepare anti-diabetic preparations. On the other hand, there is no study of plants currently used to treat diabetes in Sri Lankan Siddha Medicine. Hence, the aim of this study is to identify and document the plant species currently used in anti-diabetic preparations in Eastern Province, also enabling a comparative analysis with historical uses. Further, assessing the level of scientific evidence (*in vitro, in vivo*, and clinical studies) available for recorded species. A systematically prepared questionnaire was used to conduct an ethnobotanical survey with 27 Siddha healers residing in Eastern Province to identify the currently used anti-diabetic plants. Furthermore, Web of Science electronic database was used to assess the level of scientific evidence available excluding widespread and very well studied species. On average 325 diabetic patients were seen by 27 healers per week. Interestingly, inorganic substances, and animal parts used as ingredients in historical anti-diabetic preparations are currently not used in Eastern Province. A total of 88 plant species from 46 families were reported in this study. *Syzygium cumini* (L.) Skeels was the most frequently recorded species and the largest number of taxa are from Fabaceae. Remarkably, one third of reported species were not stated in Sri Lankan Siddha historical documents. The highest number of plant species (59%) have been studied up to an *in vivo* level followed by no scientific evidence for anti-diabetic activity found (27%), clinical evidence (10%), and *in vitro* (2%). This is the first ethnobotanical study of plants used to treat diabetes by Siddha healers in the Eastern Province in Sri Lanka. Moreover, awareness should be created to the diabetics about the side effects of herb-drug interactions and complications caused by taking both herbal preparations and biomedical drugs.

## Introduction

There are an estimated 1.16 million people (20–79 years old) with diabetes in Sri Lanka with 0.60 million undiagnosed cases. Furthermore, in 2015 16,319 (20–79 years old) deaths caused by diabetes were recorded (IDF, 2015). About 70% of rural population relies on traditional medicinal system as their primary health care (Perera, 2012). While there are two types of diabetes: Type 1 (insulin deficiency) and type 2 (insulin resistance), globally the majority of cases are type 2 diabetes. Heart attacks, lower limb amputation, blindness, and kidney failure are mostly caused by diabetes. The number of diabetics is rising fast in middle- and low-income countries (WHO, 2017).

Sri Lanka is a South Asian island situated in the Indian Ocean. It has 65,610 km^2^ area with a population of 21.2 million (Department of Census and Statistics Sri Lanka, 2017). The largest (75%) ethnic group is Sinhalese, followed by Sri Lankan Tamil (11%), Sri Lanka Moor (9%), and Indian Tamil (4%). The official and national languages of Sri Lanka are Sinhala and Tamil. The major and official religion is Buddhism (70%) followed by Saivism (13%), Islam (10%), and Christianity (7%) (Department of Census and Statistics Sri Lanka, 2012). There are three climatic zones (dry, intermediate, and wet) in Sri Lanka (Department of Meteorology Sri Lanka, 2016).

A total of 4,143 plant species of 214 families identified in Sri Lanka were listed in a work by Senaratna (2001). Furthermore, 75% of these species were indigenous whereas 25% of them were introduced/exotics, with 32% having become naturalized and 68% being under cultivation (Senaratna, 2001). In 2010 nearly 29.7% of Sri Lankan land area is covered by natural forests (Forest Department Sri Lanka, 2017). The vegetation of Sri Lanka can be categorized as follows according to the impact of soil and elevation: montane, sub montane, lowland rain, moist monsoon, dry monsoon, riverine dry, sparse and open, and mangrove forests (Forest Department Sri Lanka, 2017). The most frequently identified plant families in the montane forests were Lauraceae (*Actinodaphne, Cinnamomum*, and *Litsea*) and Myrtaceae (*Eugenia, Rhodomyrtus, and Syzygium*) (Werner and Balasubramaniam, 1992). The main Sri Lankan export crops are *Camellia sinensis* (tea), *Hevea brasiliensis* (rubber), and *Cocos nucifera* (coconut) (Department of Census and Statistics Sri Lanka, 2017).

Four traditional medicinal systems (Ayurveda, Siddha, Unani, and Deshiya Chikitsa) are currently practiced in Sri Lanka (Weragoda, 1980). Siddha (or Tamil) Medicine is mostly practiced in the Eastern and Northern Provinces of Sri Lanka (Sivashanmugarajah, 2000). So far, only two ethnobotanical surveys have been carried out in Jaffna (Rajamanoharan, 2014) and Vavuniya (Rajamanoharan, 2016), Northern Province. However, so far, no ethnobotanical survey has been carried out in Eastern Province. A recent review of Sri Lankan Siddha historical documents revealed 171 species in 73 families were used to treat diabetes in Sri Lankan Siddha Medicine (Sathasivampillai et al., 2017). However, there is no documentation of species currently used to treat diabetes in Sri Lankan Siddha Medicine.

For centuries a large number of disorders including diabetes have been managed and treated using traditional herbal preparations (Nearing, 1985). Ethnobotanical surveys focusing on the use of medicinal substances play an important role in safeguarding and understanding local/traditional and local knowledge and are seen a starting point for drug discovery (Fabricant and Farnsworth, 2001). Safeguarding is, however, only one element of such research. Modern use of “traditional” medicines are embedded in complex expectations both by those who are the keepers of this knowledge and by the wider society. However, outcomes of ethnobotanical surveys are not frequently compared with the available published scientific reports to study the pharmacological properties of plants used in the preparations. Recent surveys on anti-diabetic plants have been published for many countries such as India (Vidyasagar and Siddalinga, 2013), South Africa (Davids et al., 2016), Nigeria (Salihu Shinkafi et al., 2015), México (Andrade-Cetto and Heinrich, 2005), Turkey (Durmuşkahya and Öztürk, 2013), and China (Guo et al., 2017).

Sri Lankan local and traditional medicine is a practice which has developed as a part of the historical development of Sri Lanka incorporating not only autochthonous traditions but also the multiple impacts of diverse cultures. Our previous research (Sathasivampillai et al., 2017) identified a large number of taxa documented in historical texts which were a part of the formal curriculum for training Siddha medical practitioners for conditions associated with diabetes.

Therefore, the aim of this work is to identify and document the species currently used to treat diabetes in Eastern Province, Sri Lanka in Siddha Medicine, enabling a comparative analysis with the historical uses. With this we want to contribute to an understanding of continuity and change of practice as it relates to diabetes in this culture. This also forms a basis for a systematic, comparison with medicinal plants usage in other regions of Sri Lanka. In addition, we assess the levels of scientific evidence (*in vitro, in vivo*, and clinical studies) of the reported species based on a bibliographic assessment.

## Background and methods

### The study region

**Figure 1:**
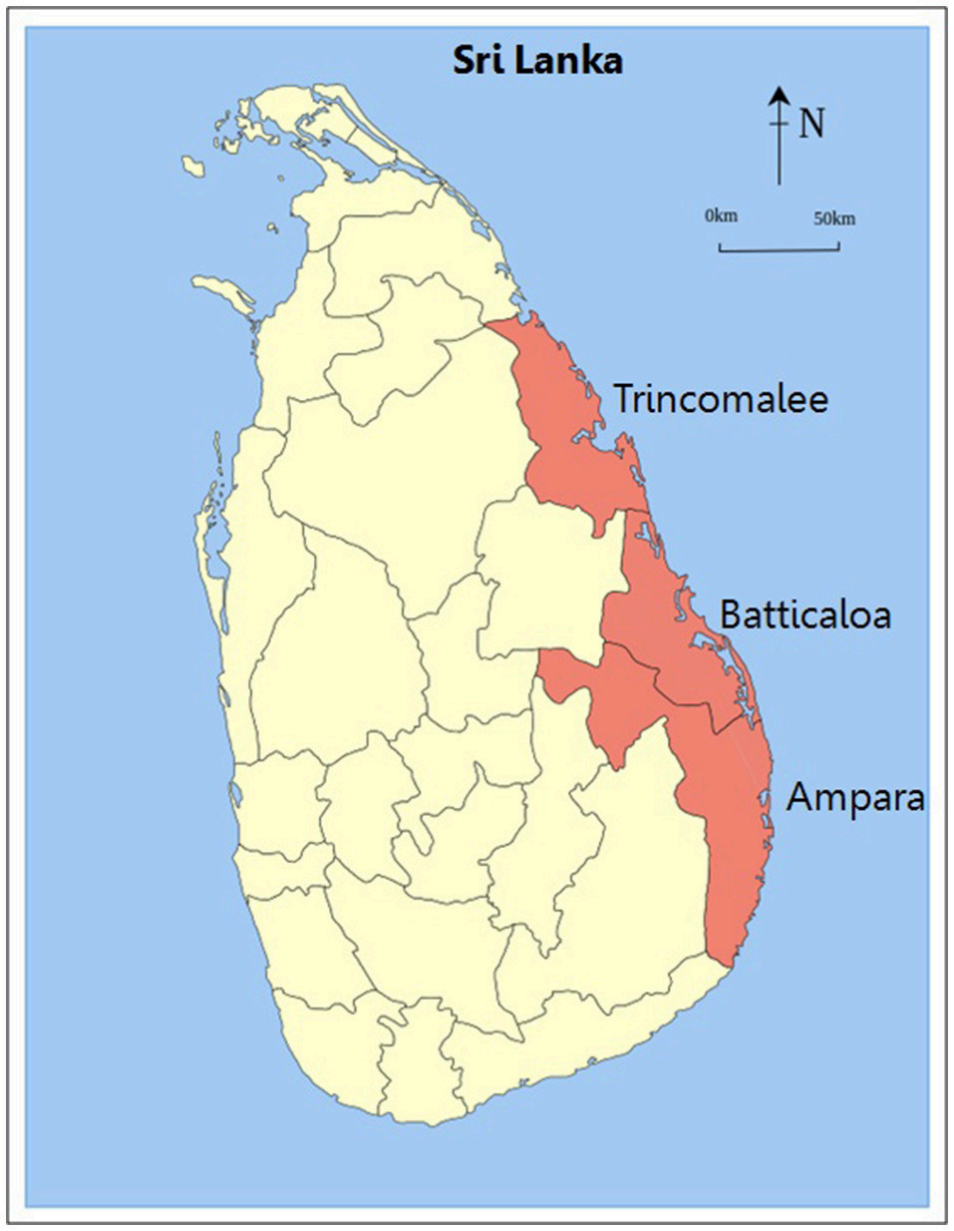
Map of study region based on https://en.wikipedia.org.

This ethnobotanical study was conducted with Siddha healers residing in the Eastern Province in the dry climatic zone of Sri Lanka which consists of three districts (Batticaloa, Ampara, and Trincomalee) (Figure 1). Tamil is the major language spoken in this area. Siddha Medicine is mostly practiced in Tamil speaking regions around the world (AYUSH, 2016).

### Ethical approval of the research

Research ethical approval was obtained from UCL Research Ethics Committee (9141/001) on 13.06.2016 prior starting the investigation in Sri Lanka. The purpose of this study and informed consent page were read to each healer before beginning the interview. The interview was carried out only after receiving the verbal consent from each informant. Healers participated voluntarily in this study, were free to withdraw at any point in time and no compensation was provided.

This study was conducted recognizing the relevant obligations under the Convention on Biological Diversity and subsequent agreements, most notably the Nagoya Protocol which had at the time of writing (07.01.2018) not yet been signed or ratified by Sri Lanka, while the UK ratified it on 22.02.2016 (https://www.cbd.int/abs/doc/protocol/nagoya-protocol-en.pdf 07.01.2018). Also, the right to authorship and use the traditional knowledge of all informants were preserved. Using this information except scientific publication requires permission from the traditional owners of knowledge. However, no permission is required from the government to collect and preserve plant material samples within Sri Lanka. It was started prior to the publication of the ConsEFS statement on best practice in ethnopharmacological research (Heinrich et al., 2018), but has followed these guidelines.

### Ethnobotanical data collection and interviews

This ethnobotanical investigation was conducted from July to September 2016. SVS conducted the interviews jointly with PRR (community health medical officer at Planning Unit, Eastern Provincial Department of Indigenous Medicine and in charge of Eastern Provincial Herbal Garden, Trincomalee) being present during the interviews.

The interviews were carried out in Tamil. Only Siddha healers whose families have been practicing Siddha Medicine for at least two generations and are registered at the Sri Lanka Ministry of Indigenous Medicine were included in this study. Siddha healers were actively chosen by the community health medical officer from the database of registered healers. Permission and appointments were obtained verbally from each healer by the community health medical officer. Then, the interviews were held at Siddha healers' homes.

Initially, 33 Siddha healers were approached. However, 6 of them do not practice any treatment for diabetes. Hence, they were excluded from this study. Interviews using a questionnaire including semi-structured questions were conducted with the healers (Appendix). The questions focused on social and demographic data such as gender, age, and experience and species currently used to treat diabetes. With each healer, the interview lasted for a minimum of 15 minutes.

### Voucher specimens and plant identification

Reported species which are available locally or cultivated were collected. The fieldwork for collecting plant part samples was conducted during September 2016 to June 2017, a period ideal for collecting flowering and fruiting specimens. Specimens are deposited at the Herbarium of the Eastern Provincial Herbal Garden, Trincomalee (for voucher specimen numbers see Table 1) and identified by PRR. Siddha healers mentioned Tamil names of the species. Scientific names and families are based on Sugathadasa et al. (2008) and validated using The Plant List (2013) and the Royal Botanic Gardens Kew, Medicinal Plant Service (2018).

**Table 1:** Demographic data of the Siddha healers (*N* = 27).

**Category**	**No. of healers**	**Percentage/%**
**GENDER**		
Male	25	93
Female	2	7
**AGE/YEAR**		
21–30	0	0
31–40	6	22
41–50	0	0
51–60	0	0
61–70	17	63
71–80	3	11
81–90	1	4
90–100	0	0
**EXPERIENCE/YEARS**		
1–10	1	4
11–20	5	19
21–30	0	0
31–40	0	0
41–50	17	63
51–60	3	11
60–70	1	4
Total	27	100

### Data analysis

A database of reported species including family, scientific name, part used, and number of participants citing it as being of medicinal importance was created. Plant species currently used to treat diabetes by Siddha healers (this study) were compared with the species historically used to treat diabetes in Sri Lankan Siddha Medicine (Sathasivampillai et al., 2017). Also, species confirmed in this work were compared with the species used to treat diabetes reported in the ethnobotanical studies previously carried out in the other areas where Siddha Medicine is mostly practiced in Jaffna (Rajamanoharan, 2014) and Vavuniya (Rajamanoharan (2016). Other comparisons with ethnobotanical information were carried out based on local knowledge of Eastern Province of Sri Lanka by the authors SVS and PRR. Furthermore, an assessment of the levels of scientific evidence of each species was carried out using the electronic database Web of Science until September 2017. The method described in Sathasivampillai et al. (2017) was followed to identify the relevant published studies. Species listed in Brendler (2010), Upton et al. (2016), American Herbal Pharmacopoeia (2017), European Medicines Agency (2018), and WHO (1999, 2004, 2007, 2009) were considered to be very well studied and global plants. Therefore, they were excluded from the literature search to identify the level of scientific evidence.

## Results and discussion

### Socio-demographic characteristics of the siddha healers participated in this study

A total of 27 Siddha healers residing in Eastern Province and currently treating diabetes were interviewed in detail for this study. The majority of participants were men. The highest number of participants were in the 61–70 age group. Also, the majority of the healers had 41–50 years of experience practicing Siddha Medicine (Table 1).

### Current diagnosis methods employed by siddha healers

This study relies on the self-reporting by the healers on their practice. While some of the cases of diabetes treated may well be confirmed using bio-medical diagnosis, it was not possible in the context of this study to ascertain any diagnosis. Diabetes is termed as Neerilivu (
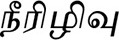
) in Siddha Medicine (Anonymous, 2000). The common symptoms of diabetes in Siddha Medicine mentioned include dry tongue, chest and throat; feeling lazy, ants and flies gather around the urine, weight loss, feeling thirsty, and excessive urination (Anonymous, 2000, 2003). Diabetes is categorized into 24 types referring to the color and taste of the urine in Siddha Medicine (Sithamparthanuppillai, 1982). In Siddha Medicine eight diagnostic methods (pulse examination, touch, tongue examination, body color, speech, eye, stool, and urine) are used to diagnose a disorder (Narayanaswami, 1975). However, in this investigation Siddha healers mentioned they only use pulse reading to diagnose diabetic cases combination with above mentioned Siddha symptoms of diabetes.

### Number of diabetic patients seen by a siddha healer

According to the information provided by the Siddha healers, on average 12 diabetics are seen by a healer per week. However, they did not show any written evidences of number of patients consulting them. Also, they did not have any information of how many of the patients come back for a regular consultation. Usually preparations sufficient for 1 month was given to each patient. Anti-diabetic preparations are prepared by the healers at home.

### Types of ingredients currently used

Herbal, inorganic (such as metals and minerals), and animal products have been recorded commonly as elements of Siddha Medicine (NIS, 2018). Also, these three types of ingredients were included in Siddha historical documents analyzed for anti-diabetic preparations (Sathasivampillai et al., 2017). Siddha healers in this study reported only botanical ingredients to prepare anti-diabetic preparations.

### Species reported by siddha healers

Overall, 88 species from 46 families was documented in this study (Table 2). *Syzygium cumini* was the most cited species (cited by 21 Siddha healers) followed by *Gymnema sylvestre, Artocarpus heterophyllus, Salacia reticulata*, and *Achyranthes aspera*. The largest number of reported taxa are from the Fabaceae. Leaves were cited as the most frequently used plant part followed by fruits, whole plant, root, and bark. The majority of the plants documented in this study were South Asian medicinal plants, like *Withania somnifera, Typhonium trilobatum, Tribulus terrestris, Toddalia asiatica*, and *Tinospora sinensis* followed by food plants including *Achyranthes aspera, Borassus flabellifer, Cinnamomum verum, Eleusine coracana*, and *Limonia acidissima*.

**Table 2:** Reported plant species used to treat diabetes in Siddha Medicine in Eastern Province (*n* = 27).

**Family, scientific name, voucher specimen identification**	**Tamil name**	**Part used**	**Local use, number of times cited**		
			**Food**	**Medicine**	**Other**
**ACANTHACEAE**					
*Hygrophila auriculata* (Schumach.) Heine	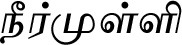 (Neermulli)	Leaf		4	
*Andrographis paniculata* (Burm.f.) Nees (VS006)^*^^#^	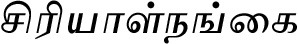 (Siriyaalnangai)	Whole plant		3	
**AMARANTHACEAE**					
*Achyranthes aspera* L. (VS001)	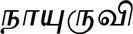 (Naayuruvi)	Whole plant	11		
*Aerva lanata* (L.) Juss. (VS003)^#^	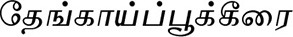 (Thengaaippookkeerai)	Whole plant		2	
*Alternanthera sessilis* (L.) R.Br. ex DC. (VS004)	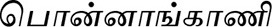 (Ponnaangkaani)	Leaf	2		
**AMARYLLIDACEAE**					
*Allium sativum* L.^*^	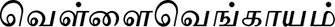 (Vellai vengaayam)	Bulb	3		
**ANACARDIACEAE**					
*Anacardium occidentale* L. (VS005)^*^	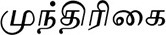 (Munthirihai)	Fruit	1		
**APIACEAE**					
*Anethum graveolens* L.^*^	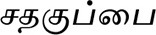 (Sathahuppai)	Seed		1	
*Coriandrum sativum* L.^*^^#^	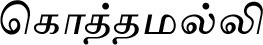 (Koththamalli)	Seed	5		
*Cuminum cyminum* L.^*^	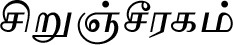 (Sirunjcheeraham)	Fruit	5		
*Ferula assa-foetida* L.^*^	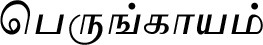 (Perungkaayam)	Resin	1		
*Foeniculum vulgare* Mill.^*^	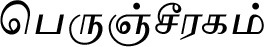 (Perunjcheeraham)	Fruit	2		
*Trachyspermum roxburghianum* (DC.) H. Wolff^$^	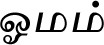 (Omam)	Fruit		3	
**APOCYNACEAE**					
*Catharanthus roseus* (L.) G.Don (VS013)^*^^#^	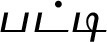 (Patti)	Root		1	
*Gymnema sylvestre* (Retz.) R.Br. ex Sm. (VS024)^*^^#^	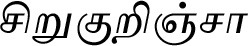 (Sirukurinjaa)	Leaf, root	18		
**ARACEAE**					
*Typhonium trilobatum* (L.) Schott^#^^$^	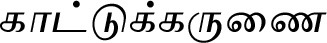 (Kaattukkarunai)	Rhizome		1	
**ARECACEAE**					
*Borassus flabellifer* L.	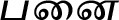 (Panai)	Fruit	2		
**ARISTOLOCHIACEAE**					
*Aristolochia bracteolata* Lam.^#^	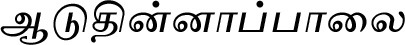 (Aaduthinnaappaalai)	Whole plant		1	
**ASCLEPIADACEAE**					
*Calotropis procera* (Aiton) Dryand.^#^	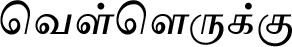 (Vellerukku)	Root		1	
*Dregea volubilis* (L.f.) Benth. ex Hook.f. (VS018)^#^	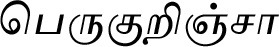 (Perukurinjaa)	Leaf	4		
**ASTERACEAE**					
*Cyanthillium cinereum* (L.) H.Rob. (VS016)	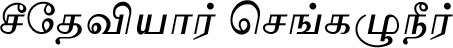 (Seetheviyaar sengkaluneer)	Whole plant		2	
*Eclipta prostrata* (L.) L. (VS019)	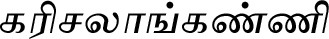 (Karisalaangkanni)	Whole plant		3	
**BASELLACEAE**					
*Basella alba* L. (VS009)^#^^$^	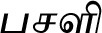 (Pasali)	Leaf	3		
**BIGNONIACEAE**					
*Stereospermum chelonoides* (L.f.) DC.	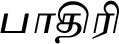 (Paathiri)	Root		2	
**CALOPHYLLACEAE**					
*Mesua ferrea* L.^$^	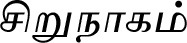 (Sirunaaham)	Flower		2	
**CAPPARACEAE**					
*Crateva adansonii* DC.^#^^$^	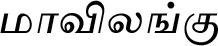 (Maavilangu)	Bark		1	
**CARICACEAE**					
*Carica papaya* L. (VS011)^*^^#^	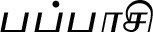 (Pappaasi)	Leaf	3		
**CELASTRACEAE**					
*Salacia reticulata* Wight (VS036)	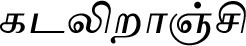 (Kadaliraanji)	Bark	12		
**COMBRETACEAE**					
*Terminalia arjuna* (Roxb. ex DC.) Wight & Arn. (VS041)^*^	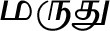 (Maruthu)	Bark			1
*Terminalia bellirica* (Gaertn.) Roxb. (VS042)^*^	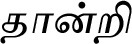 (Thaandri)	Fruit		2	
*Terminalia chebula* Retz.^*^	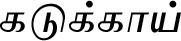 (Kadukkaai)	Fruit		5	
**CONVOLVULACEAE**					
*Evolvulus nummularius* (L.) L. (VS020)^#^^$^	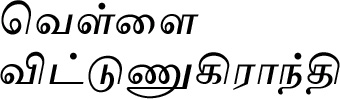 (Vellai vittunukiraanthi)	Whole plant		1	
*Ipomoea aquatica* Forssk. (VS025)	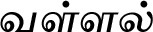 (Vallal)	Leaf	4		
*Merremia emarginata* (Burm. f.) Hallier f.^#^	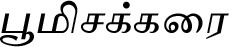 (Poomisakkarai)	Rhizome		3	
**COSTACEAE**					
*Cheilocostus speciosus* (J.Koenig) C.D.Specht (VS014)	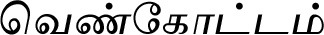 (Venkottam)	Rhizome		3	
**CUCURBITACEAE**					
*Coccinia grandis* (L.) Voigt (VS015)	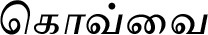 (Kovvai)	Leaf	9		
*Momordica charantia* L. (VS027)^*^	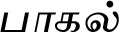 (Paahal)	Fruit, leaf	3		
*Mukia maderaspatana* (L.) M.Roem. (VS028)	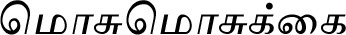 (Mosumosukkai)	Leaf	6		
**FABACEAE**					
*Cassia fistula* L. (VS012)^*^	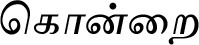 (Kondrai)	Bark		1	
*Indigofera aspalathoides* DC.^#^^$^	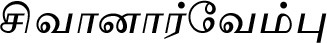 (Sivanaarvembu)	Whole plant		2	
*Indigofera tinctoria* L.^$^	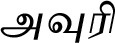 (Avuri)	Root		2	
*Pongamia pinnata* (L.) Pierre (VS035)^#^^$^	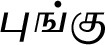 (Pungu)	Root		3	
*Senna auriculata* (L.) Roxb. (VS037)	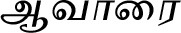 (Aavaarai)	Bark, flower, leaf, root, seed		5	
*Senna sophera* (L.) Roxb. (VS038)	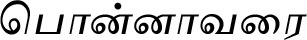 (Ponnaavarai)	Flower	2		
*Sesbania grandiflora* (L.) Pers. (VS039)^#^	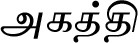 (Ahaththi)	Leaf	3		
*Trigonella foenum- graecum* L.^*^	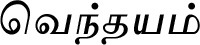 (Venthayam)	Seed	6		
**LAMIACEAE**					
*Ocimum tenuiflorum* L. (VS030)^*^^#^	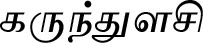 (Karunthulasi)	Whole plant		3	
**LAURACEAE**					
*Cinnamomum verum* J.Presl^*^	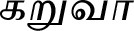 (Karuvaa)	Bark, leaf	1		
**MALVACEAE**					
*Abutilon indicum* (L.) Sweet	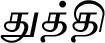 (Thuththi)	Whole plant		3	
*Thespesia populnea* (L.) Sol. ex Corrêa (VS043)	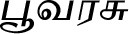 (Poovarasu)	Bark			1
**MELIACEAE**					
*Azadirachta indica* A.Juss. (VS008)^*^	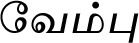 (Vembu)	Bark, tender, leaf		4	
**MENISPERMACEAE**					
*Coscinium fenestratum* (Goetgh.) Colebr.	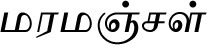 (Maramanjal)	Stem		4	
*Tinospora sinensis* (Lour.) Merr.^*^	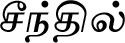 (Seenthil)	Stem		1	
**MORACEAE**					
*Artocarpus heterophyllus* Lam. (VS007)	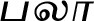 (Palaa)	Mature	13		
*Ficus benghalensis* L. (VS021)	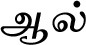 (Aal)	Bark			2
*Ficus racemosa* L. (VS022)	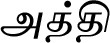 (Aththi)	Bark		2	
*Ficus religiosa* L. (VS033)	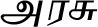 (Arasu)	Bark			3
**MYRISTICACEAE**					
*Myristica fragrans* Houtt.	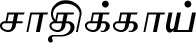 (Saathikkaai)	Leaf, mace	3		
**MYRTACEAE**					
*Syzygium aromaticum* (L.) Merr. & L.M.Perry^*^	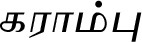 (Karaambu)	Flower, bud	4		
*Syzygium cumini* (L.) Skeels (VS040)	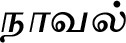 (Naaval)	Bark, root, seed	2		
**NYCTAGINACEAE**					
*Boerhavia diffusa* L.^#^	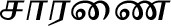 (Saaranai)	Leaf		2	
**OXALIDACEAE**					
*Averrhoa carambola* L.^#^	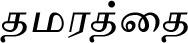 (Thamaraththai)	Fruit	1		
**PASSIFLORACEAE**					
*Passiflora edulis* Sims (VS032)^*^^#^	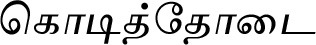 (Kodiththodai)	Leaf	2		
**PEDALIACEAE**					
*Pedalium murex* L.^#^^$^	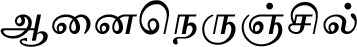 (Aanainerunjil)	Whole plant		2	
**PHYLLANTHACEAE**					
*Phyllanthus emblica* L. (VS033)^*^	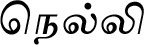 (Nelli)	Fruit, root	8		
**PIPERACEAE**					
*Piper longum* L. (VS034)^*^	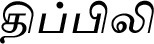 (Thippili)	Fruit		2	
*Piper nigrum* L.^*^	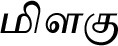 (Milahu)	Fruit	2		
**PLANTAGINACEAE**					
*Bacopa monnieri* (L.) Wettst.^*^^#^	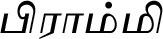 (Piraammi)	Leaf		1	
*Scoparia dulcis* L.	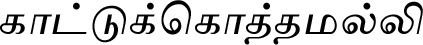 (Kaattukkoththamalli)	Leaf		1	
**POACEAE**					
*Chrysopogon zizanioides* (L.) Roberty	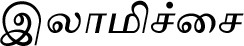 (Ilaamichchai)	Root		1	
*Cynodon dactylon* (L.) Pers. (VS017)^*^^#^	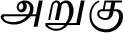 (Aruhu)	Whole plant		3	
*Eleusine coracana* (L.) Gaertn.	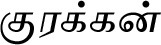 (Kurakkan)	Seed	1		
*Oryza sativa* L. (VS031)^*^	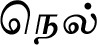 (Nel)	Seed	4		
*Setaria italica* (L.) P.Beauv.^#^	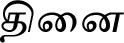 (Thinai)	Seed	2		
**RANUNCULACEAE**					
*Nigella sativa* L.^*^	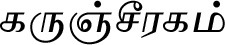 (Karunjcheeraham)	Seed		2	
**RUBIACEAE**					
*Pavetta indica* L.^#^^$^	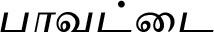 (Paavattai)	Leaf		2	
**RUTACEAE**					
*Aegle marmelos* (L.) Corrêa (VS002)^*^	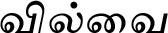 (Vilvai)	Bark, fruit	7		
*Limonia acidissima* Groff (VS026)^$^	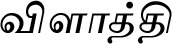 (Vilaaththi)	Fruit	2		
*Murraya koenigii* (L.) Spreng. (VS029)^*^	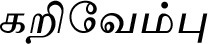 (Karivembu)	Leaf	10		
*Toddalia asiatica* (L.) Lam.^*^^#^	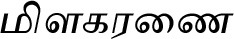 (Milaharanai)	Root		1	
**SAPINDACEAE**					
*Cardiospermum halicacabum* L. (VS010)	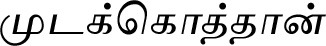 (Mudakkoththaan)	Leaf	5		
**SCHISANDRACEAE**					
*Illicium verum* Hook.f.^*^^#^	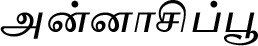 (Annaasippoo)	Fruit	1		
**SOLANACEAE**					
*Withania somnifera* (L.) Dunal^*^	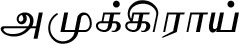 (Amukkiraai)	Rhizome		4	
**ZINGIBERACEAE**					
*Curcuma longa* L.^*^	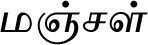 (Manjal)	Rhizome	5		
*Elettaria cardamomum* (L.) Maton^*^	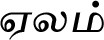 (Elam)	Fruit	5		
*Kaempferia galanga* L.^$^	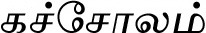 (Kachcholam)	Rhizome		2	
*Zingiber officinale* Roscoe^*^	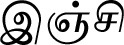 (Inji)	Rhizome	4		
**ZYGOPHYLLACEAE**					
*Tribulus terrestris* L. (VS044)^*^	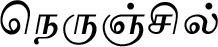 (Nerunjchil)	Whole plant		1	

*Local use, Food, medicinal, and other uses of plant species were categorized according to the most common local use of the particular plant species*.

* *Plant species excluded from further analysis in this study*.

# *Plant species had not been reported in either anti-diabetic preparations in Sri Lankan Siddha historical documents or ethnobotanical surveys carried out in Siddha Medicine practicing regions in Sri Lanka*.

$ *Plant species without any bioscientific evidence for anti-diabetic activity*.

Remarkably, one third of the species reported in this study had not been recorded before either in anti-diabetic preparations of Sri Lankan Siddha historical documents or in the ethnobotanical surveys carried out in regions of Sri Lanka where Siddha Medicine is practiced (marked as “#” in Table 2). For example, *Sesbania grandiflora* and *Pedalium murex* are reported for the first time in this study as ingredients in Siddha anti-diabetic preparations in Sri Lanka. At the same time there is a strong overlap with the historical Siddha documents with 61% of the recorded species also stated as anti-diabetic preparations including *Ficus racemosa* and *Salacia reticulata*. Since these are used as textbooks for training Siddha healers this indicates that both written records (Leonti, 2011) and oral transmission / local innovations form the basis for the species used. The most frequently used species in this study (*S. cumini*) was also used in the anti-diabetic preparations in the historical documents. *Senna auriculata* is the most frequently stated in the historical preparations used to treat diabetes in Sri Lankan Siddha Medicine (Sathasivampillai et al., 2017) but only five healers in this work consider it to be a useful anti-diabetic therapy.

A total of eight species were recorded previously to treat diabetes in Northern Province. Seven species were reported in Vavuniya and only *Scoparia dulcis* was recorded in Jaffna (Rajamanoharan, 2014). Species such as *Momordica charantia* and *G. sylvestre* were reported in both Eastern and Northern Provinces. Also, species such as *Andrographis paniculata* and *Toddalia asiatica* had been only confirmed in Eastern Province. Interestingly, the most frequently mentioned species (*S. cumini*) in this study and cited in historical documents (*S. auriculata)* had not been recorded in Northern Province (Rajamanoharan, 2014, 2016; Sathasivampillai et al., 2017).

Side effects and toxicity studies of plant extracts and herbal preparations play a very important role in assessing the safety and efficacy of drugs purposes. Some species are clearly toxic, like *Aristolochia bracteolata* (cf. Michl et al., 2016) and their use cannot be endorsed. More complex is the situation with regards to potential herb-drug interactions. However, a detailed analysis of such interactions would be limited by the evidence for specific interactions, for example, with the multiple steps of the detoxification system of xenobiotics that could be affected and the specific composition of the preparation which might be active. This therefore, should be the focus of specific studies of the most important plants using well-characterized extracts.

### Levels of scientific evidence of reported species

Based on the Web of Science electronic database of the pharmacological evidence linked to anti-diabetic activities of the species were assessed [excluding 39 (44%) very well studied and globally distributed species, marked with an “^*^” in Table 2]. The levels of scientific evidence were established, including information on the plant part used, active extract or compound, model, dose, and duration for the remaining 49 (56%) species (Appendix). Four levels of scientific evidence were established:

1. There is no reported bioscientific evidence
2. *In vitro* evidence only
3. *In vivo* evidence and active compound identified and
4. Clinical evidence and active compound identified.

#### Species with no bioscientific evidence

There was no scientific evidence for anti-diabetic activity found for 13 species (27%). Hence, Anti-diabetic and toxicity studies should be carried out in the future focusing on the most frequently used species such as *Limonia acidissima, Crateva adansonii, Evolvulus nummularius, P. murex*, and *Mesua ferrea*.

#### *In vitro* evidence reported

Only for *Mukia maderaspatana* and *Setaria italica in vitro* bioassays form the basis for as an evidence base. Ethanol extract of *S. italica* seeds showed inhibitory activity in the α-glucosidase inhibition assay with an IC_50_ 1.1 to 1.4 μg/ml (Kim et al., 2011). The active compounds have so far not been isolated from either of these species calling for further phytochemical studies, especially for *S. italica* (foxtail millet) seeds since these had higher α-glucosidase inhibitory activity at a low dose (IC_50_ 1.1 to 1.4 μg/ml). This species would also be of particular interest because of its wide distribution and use as a (specialist) food.

#### *In vivo* evidence and active compound found

The majority (29 out of 49, 59%) have *in vivo* evidence including *Coccinia grandis, Sesbania grandiflora, Cardiospermum halicacabum, Thespesia populnea*, and *Coscinium fenestratum*. Compounds with anti-diabetic activity have been isolated from eight species (*Eclipta prostrata, Cheilocostus speciosus, S. auriculata, F. benghalensis, Myristica fragrans, S. cumini, Averrhoa carambola*, and *S. dulcis*) studied in *in vivo* models. Costunolide (5 mg/kg) isolated from *C. speciosus* roots was orally administered to Streptozotocin induced diabetic rats daily for 30 days. This treatment significantly reduced blood glucose concentration (Eliza et al., 2009b). In another study 2-(3-acetoxy-4,4,14-trimethylandrost- 8-en-17-yl) (5 mg/kg) identified in *S. auriculata* flowers orally administered to Alloxan induced diabetic rats for 15 days decreased elevated blood glucose levels (Venkatachalam et al., 2013). Further phytochemical, *in vivo*, clinical, and toxicity studies should be carried out to identify the active compounds and further evaluate the anti-diabetic activities of the species with reported *in vivo* evidence. Species such as *Borassus flabellifer, Alternanthera sessilis, Ipomoea aquatica, Senna sophera*, and *Chrysopogon zizanioides* are potential candidates for clinical studies.

#### Clinical evidence and active compound found

Clinical evidence is available for 5 out of 49 species (10%): *Cyanthillium cinereum, S. reticulata, Artocarpus heterophyllus, Eleusine coracana*, and *F. racemosa*. So far anti-diabetic compounds have only been isolated from *F. racemosa*, but these compounds have not been studied in clinical trials. *F. racemosa* bark water extract (1.2 g/d) orally administered to type 2 diabetic patients (18 men and 12 women) for 1 month showed 15% of reduction of fasting blood glucose levels and 22% of reduction of postprandial blood glucose levels (Ahmed et al., 2011). However, the authors did not state whether this clinical trial was a controlled trail or not. In another study *F. racemosa* bark extract (100 mg twice a day) was administered orally to diabetics (25 male and 25 female) for 15 days and reduced serum glucose concentrations (Gul-e-Rana et al., 2013).

## Conclusion

This is the first ethnobotanical study of plants used to treat diabetes by Siddha healers in the Eastern Province in Sri Lanka. This study aimed at documenting and comparing the current ethnobotanical knowledge from the Siddha healers linked to the treatment of diabetes with plant species recorded in the historical documents and in the few other studies which exist on Siddha medicine. Overall, the bioscientific evidence is limited and priority should be given to the most widely used species. While we excluded globally distributed species, some of them have a better evidence base in terms of safety and pharmacology / clinical effectiveness, and thus they may be better suited in primary health care projects. With this medical tradition's importance also as an element of primary health care, scientific evidence is needed first and foremost on the Siddha medicines' safety and lack of toxicity [e.g., *Aristolochia* species like *A. bracteolate* clearly not be endorsed as a phytomedicine (Michl et al., 2016)]. This study demonstrates that there is a wealth of knowledge among Siddha healers about managing diabetes, and that there is an urgent need for more studies providing a better evidence-base for these uses. In many cases the chemistry of the species is known relatively well and thus further bibliographic assessments can form a starting point for such an assessment. In further steps observational and ideally intervention studies are essential.

Ethnobotanical surveys should be carried out in the other regions of Sri Lanka for documenting the useful species utilized by Siddha healers before this knowledge may disappear in the future. Importantly, as many diabetics are currently taking combination of herbal preparations with biomedical medications such as Metformin, ethnobotanical surveys should be carried out with diabetics to create awareness of potential herb-drug interactions, side effects and complications caused by taking both biomedical and traditional medicinal preparations.

Furthermore, the Sri Lankan government also needs to advice traditional healers, biomedical doctors, and the public on the species safety and potential uses. Also, potential interactions between “Western” and traditional medicinal preparations need to be assessed and then should communicated to a wider public. Last but not least, this work provides new opportunities to discover novel compounds which could be used as active compounds in future drug discovery.

## Author contributions

The first author SS has contributed 50% and the rest 50% have been equally contributed by both authors PR and MH to this work.

### Conflict of interest statement

The authors declare that the research was conducted in the absence of any commercial or financial relationships that could be construed as a potential conflict of interest.
