# Supplementary material for: Siddha Medicine in Eastern Sri Lanka Today–Continuity and Change in the Treatment of Diabetes
Source: Front Pharmacol. 2018 Oct 10;9:1022. doi: 10.3389/fphar.2018.01022 (PMC6192459; doi:10.3389/fphar.2018.01022)
Supplement: Supplementary file 1 [file Data_Sheet_1.pdf]

Appendix
Questionnaire
Section A
1. Did any diabetic patients consult you?
(i) Yes (ii) No

2. How many diabetic patients consult you in a week?

3. Name the preparations you use to treat diabetes?

4. Describe the procedures for preparing the antidiabetic preparations in detail?

5. How do you diagnose diabetes?

6. What are the plant species and parts do you use in the antidiabetic preparations?

7. What are the animals and animal ingredients do you use in antidiabetic preparations?

8. What are the inorganic substances such as borax and mercury do you use in antidiabetic preparations?

9. Where do you obtain the raw materials such as the plant parts, animal ingredients, and inorganic substances to prepare the antidiabetic preparations?

Section B
In order to compare the responses, we need some general demographic data about yourself.
10. Age:

11. Gender: Male / Female

12. The number of years of experience in practicing Siddha Medicine:

Table 1
Pharmacological studies related to anti-diabetic effects of reported species (except very well studied and globally distributed species)

Family, scientific name, level of evidence	Part used	Active extract / compound	Bioassay / model	Dose	Duration	Reference
**Acanthaceae**						
Hygrophila auriculata (Schumach.) Heine						
In vivo	Aerial	Ethanol	SID	100 mg/kg bw	3 week	Vijayakumar et al. (2006)
**Amaranthaceae**						
Achyranthes aspera L.						
In vivo	Leaf, stem	Ethanol	AID	200 mg/kg	3 week	Talukder et al. (2012)
In vivo	Whole plant	Methanol, Aqueous	AID	2 g/kg	7 d	Akhtar and Iqbal (1991)
Aerva lanata (L.) Juss.						
In vitro	Whole plant	70% Ethanol	AGR	108.7 µg/ ml	NA	Riya et al. (2015)
In vitro	Whole plant	Ethyl acetate	AGR	208.04 µg/ml	NA	Riya et al. (2015)
In vitro	Whole plant	70% Ethanol	AGI	81.76 µg/ml	NA	Riya et al. (2015)
In vitro	Whole plant	Ethyl acetate	AGI	108.23 µg/ ml	NA	Riya et al. (2015)
In vivo	Aerial	50% Ethanol	AID	500 mg/kg	2 week	Vetrichelvan and Jegadeesan (2002)
In vivo	Aerial	Methanol, Aqueous	SID	200 mg/kg	2 week	Rajesh et al. (2012)
In vivo	Leaf	Ethanol	AID	400 mg/kg	28 d	Deshmukh et al. (2008)
In vivo	Root	Methanol	SNI	10 mg/kg	2 week	Agrawal et al. (2013)

Family, scientific name, level of evidence	Part used	Active extract / compound	Bioassay / model	Dose	Duration	Reference
In vivo	Whole plant	70% Ethanol	SID	500 mg/kg	300 min	Riya et al. (2015)
Alternanthera sessilis (L.) R.Br. ex DC.						
In vivo	Aerial	Ethanol	HFD and SID	250 mg/kg	2 week	Tan and Kim (2013)
**Arecaceae**						
Borassus flabellifer L.						
In vivo	Root	Ethanol	AID	100 mg/kg	4 week	Debnath et al. (2013)
**Aristolochiaceae**						
Aristolochia bracteolata Lam.						
In vivo	Whole plant	Methanol	SID	200 mg/kg	28 d	Raju and Reddy (2017)
**Asclepiadaceae**						
Calotropis procera (Aiton) Dryand.						
In vitro	Leaf	Aqueous	AGI	3.25 mg/ml	NA	Kazeem et al. (2016)
In vitro	Leaf	Ethanol	AAI	7.80 mg/ml	NA	Kazeem et al. (2016)
In vivo	Latex	NA	AID	100 mg/kg	31 d	Roy et al. (2005)
In vivo	Leaf	Aqueous, chloroform, ethanol	SID	200 mg/kg	15 d	Ismaiel Ali Abd Alrheam and Saad Al Shehri (2015)
In vivo	Latex	NA	SID	200 mg/kg	15 d	Ismaiel Ali Abd Alrheam and Saad Al Shehri (2015)
In vivo	Latex	Aqueous	AID	100 mg/kg	90 d	Kumar and Padhy (2011)
In vivo	Leaf	Ethanol	SID	300 mg/kg	4 week	Neto et al. (2013)

Family, scientific name, level of evidence	Part used	Active extract / compound	Bioassay / model	Dose	Duration	Reference
In vivo	Root	Methanol	SID	100 mg/kg	42 d	Yadav et al. (2014)
Dregea volubilis (L.f.) Benth. ex Hook.f.						
In vivo	Leaf	Ethanol	SID	200 mg/kg	210 min	Natarajan and Arul Gnana Dhas (2013)
**Asteraceae**						
Cyanthillium cinereum (L.) H.Rob						
Clinical	Root	NS	T2D person	6 g/d	6 month	Bin Sayeed et al. (2013)
Eclipta prostrata (L.) L.						
In vivo	Whole plant	Eclalbasaponin II	AID	10 mg/ml	28 d	Rahman et al. (2011)
In vivo	Whole plant	Methanol	AID	300 mg/kg	7 d	Rahman et al. (2011)
**Bignoniaceae**						
Stereospermum chelonoides (L.f.) DC.						
In vivo	Bark	Ethanol	SID	200 mg/kg	14 d	Balasubramanian et al. (2009)
**Celastraceae**						
Salacia reticulata Wight						
In vivo	Leaf	Aqueous	T1D	1 mg	30 min	Yoshino et al. (2009)
In vivo	Root	Methanol	FFD	0.4 g/100 g feed	4 week	Rafiq and Mitra (2010)

Family, scientific name, level of evidence	Part used	Active extract / compound	Bioassay / model	Dose	Duration	Reference
In vivo	Stem	Aqueous	KK-Ay	0.45 mg dry matter/ml	4 week	Im et al. (2009)
Clinical	Bark	NA	T2D	2 g/d	90 d	Radha and Amrithaveni (2009)
**Convolvulaceae**						
Ipomoea aquatica Forssk.						
In vivo	Leaf, stem	Aqueous	GCH	3.4 g/kg	2 h	Malalavidhane et al. (2000)
In vivo	Leaf, stem	Aqueous	GCH	3.3 g/kg	2 h	Malalavidhane et al. (2001)
In vivo	Leaf, stem	NA	SID	3.4 g/kg	1 week	Malalavidhane et al. (2003)
Merremia emarginata (Burm. f.) Hallier f.						
In vivo	NS	Methanol	SID	100 mg/kg	28 d	Gandhi and Sasikumar (2012)
**Costaceae**						
Cheilocostus speciosus (J.Koenig) C.D.Specht						
In vivo	Rhizome	Costunolide and eremanthin	SID	20 mg/kg	NS	Eliza et al. (2011)
In vivo	Rhizome	Hexane	SID	250 mg/kg	60 d	Daisy et al. (2008) and Eliza et al. (2011)
In vivo	Rhizome	Aqueous	SID	200 mg/kg	240 min, 14 d	Rajesh et al. (2009)
In vivo	Rhizome	Ethyl acetate, methanol	SID	400 mg/kg	60 d	Daisy et al. (2008)
In vivo	Root	Costunolide	SID	5 mg/kg	30 d	Eliza et al. (2009b)
In vivo	Root	95% Ethyl acetate	AID	300 mg/kg	4 week	Bavarva and Narasimhacharya (2008)
In vitro	Leaf	Methanol	AGI	67.5 µg/ml	NA	Perera, H.K.I., et al. (2016)
**Cucurbitaceae**						

Family, scientific name, level of evidence	Part used	Active extract / compound	Bioassay / model	Dose	Duration	Reference
Coccinia grandis (L.) Voigt						
In vivo	Leaf	Aqueous	SID	0.75 g/kg	30 d	Attanayake et al. (2015)
Mukia maderaspatana (L.) M.Roem.						
In vitro	Whole plant	Methanol	RLS	0.25 mg/ml	NA	Srilatha and Ananda (2014)
**Fabaceae**						
Senna auriculata (L.) Roxb.						
In vitro	Leaf	Aqueous	AID	200 mg/kg	10 d	Sabu and Subburaju (2002)
In vivo	Flower	Aqueous	SID	0.15 g/kg	30 d	Latha and Pari, (2003)
In vivo	Flower	Methanol	AID	0.20 g/kg	8 d	Surana et al. (2008)
In vivo	Flower	Ethanol	AID	200 mg/kg	NS	Jarald et al. (2010)
In vivo	Flower	Ethanol	AID	250 mg/kg	NS	Hatapakki et al. (2005)
In vivo	Leaf	Aqueous	SID	400 mg/kg	15 d	Gupta et al. (2010b)
In vivo	Leaf	Aqueous	SID	400 mg/kg	21 d	Gupta et al. (2009a)
In vivo	Whole plant	Aqueous	SID	250 mg/kg	28 d	Juvekar and Halade (2006)
In vivo	Whole plant	Ethanol	SID	400mg/kg	28 d	Juvekar and Halade (2006)
Senna sophera (L.) Roxb.						
In vivo	Seed	Aqueous	Diabetic	2 g	4 week	Feng (2003)
Sesbania grandiflora (L.) Pers.						

Family, scientific name, level of evidence	Part used	Active extract / compound	Bioassay / model	Dose	Duration	Reference
In vivo	Leaf	Methanol	HFD and SID	200 mg/kg	28 d	Panigrahi et al. (2016)
**Malvaceae**						
Abutilon indicum (L.) Sweet						
In vitro	Leaf	Methanol	AGI	2.45 mg/ml	NA	Adisakwattana et al. (2009)
In vivo	Leaf	Methanol	SID	500 mg/kg	2 h	Adisakwattana et al. (2009)
In vivo	Leaf, twig, root	Aqueous	SID	0.25 g/kg	2 week	Krisanapun et al. (2011)
Thespesia populnea (L.) Sol. ex Corrêa						
In vivo	Fruit	Aqueous, ethanol	AID	200 mg/kg	28 d	Belhekar et al. (2013)
**Menispermaceae**						
Coscinium fenestratum (Goetgh.) Colebr.						
In vivo	Stem	Ethanol	SNI	500 mg/kg	12 d	Shirwaikar et al. (2005); Punitha et al. (2005)
**Moraceae**						
Artocarpus heterophyllus Lam.						
In vitro	Bark	Ethanol	AGI	3.53 mg/ml	NA	Ajiboye et al. (2016)
In vitro	Bark	Ethanol	AAI	4.18 mg/ml	NA	Ajiboye et al. (2016)
In vitro	Leaf	Aqueous	AAI	0.104 mg/ml	NA	Kotowaroo et al. (2006)

Family, scientific name, level of evidence	Part used	Active extract / compound	Bioassay / model	Dose	Duration	Reference
In vivo	Leaf	Ethanol	AID	100 mg/kg	7 d	Okonkwo et al. (2015)
In vivo	Mature leaf	Aqueous	SID	20 mg/kg	5 week	Chackrewarthy et al. (2010)
Clinical	Mature leaf	Aqueous	Diabetic	20 g/kg (starting material)	1 h	Fernando et al. (1991)
Ficus benghalensis L.						
In vivo	Bark	Leucopelargonidin	DIA	NS	NS	Cherian and Augusti (1993)
In vivo	Bark	Leucopelargonidin-3-0-α-L rhamnoside	AID	100 mg/kg	30 d	Cherian and Augusti (1995)
In vivo	Bark	Ethanol	AID	NS	30 d	Singh and Gupta (2006)
In vivo	NS	Ethanol	AID	250 mg/kg	2 week	Kar et al. (2003)
Ficus racemosa L.						
In vitro	Bark	Ethanol	AAI	46.7 µg/ml	NA	Trinh et al. (2016)
In vitro	Bark	Derrone	PTP	12.6 µM	NA	Trinh et al. (2017)
In vitro	Bark	Mucusisoflavone B	PTP	2.5 µM	NA	Trinh et al. (2017)
In vitro	Bark	Isoderrone	PTP	22.7 µM	NA	Trinh et al. (2017)
In vitro	Bark	Alpinumisoflavone	PTP	21.2 µM	NA	Trinh et al. (2017)
In vitro	Bark	NA	AGI	212 µg/ml	NA	Ahmed and Urooj (2010)
In vitro	Bark	NA	BGI	239 µg/ml	NA	Ahmed and Urooj (2010)
In vitro	Bark	NA	AAI	259 µg/ml	NA	Ahmed and Urooj (2010)

Family, scientific name, level of evidence	Part used	Active extract / compound	Bioassay / model	Dose	Duration	Reference
In vivo	Bark	Ethanol	HFD and SID	200 mg/kg	2 week	Veerapur et al. (2012)
In vivo	Bark	Kaempherol, quercetin, naringenin, baicalein	SID	100 mg/kg	7 d	Keshari et al. (2016)
In vivo	Fruit	80% Ethanol	T1D	1.25 g/kg	120 min	Jahan et al. (2009)
In vivo	Fruit	Ethanol	AID	100 mg/kg	21 d	Hasan et al. (2017)
In vivo	Leaf	β-Sitosterol, stigmasterol, lanosterol	SID	100 mg/kg	7 d	Kushwaha et al. (2015)
Clinical	Bark	NS	DIA	200 mg	15 d	Gul-E-Rana et al. (2013)
Clinical	Bark	Aqueous	T2D	1.2 g/d	1 month	Ahmed et al. (2011)
Ficus religiosa L.						
In vivo	Bark	Aqueous	SID	25 mg/kg	21 d	Pandit et al. (2010)
**Myristicaceae**						
Myristica fragrans Houtt.						
In vivo	Fruit	Ethanol	CID	150 mg/kg	7 d	Arulmozhi et al. (2007)
**Myrtaceae**						
Syzygium cumini (L.) Skeels						
In vitro	Fruit	Aqueous	AGI	3.3 µg/ml	NA	Trinh et al. (2016)
In vitro	Seed	Acetone	AGI	19.5 µg/ml	NA	Shinde et al. (2008)
In vitro	Seed	Acetone	AGB	6.6 µg/ml	NA	Shinde et al. (2008)
In vitro	Seed	Acetone	AGR (MAL)	114.4 µg/ml	NA	Shinde et al. (2008)
In vitro	Seed	Acetone	AGR (SUC)	261.7 µg/ml	NA	Shinde et al. (2008)

Family, scientific name, level of evidence	Part used	Active extract / compound	Bioassay / model	Dose	Duration	Reference
In vitro	Seed	70% Ethanol	AGI	24.6 µg/ml	NA	Shinde et al. (2008)
In vitro	Seed	70% Ethanol	AGB	4.6 µg/ml	NA	Shinde et al. (2008)
In vitro	Seed	70% Ethanol	AGR (MAL)	120.9 µg/ml	NA	Shinde et al. (2008)
In vitro	Seed	70% Ethanol	AGR (SUC)	299 µg/ml	NA	Shinde et al. (2008)
In vitro	Seed	Aqueous	AAI	NS	NA	Karthic et al. (2008)
In vivo	Fruit	Aqueous, ethanol	SID	100 mg/kg	NS	Yousaf et al. (2016)
In vivo	Leaf	Ethanol	AID	250 mg/kg	3 h	Schoenfelder et al. (2010)
In vivo	Leaf	Ethanol	NOR	200 mg/kg	7 d	Oliveira et al. (2005)
In vivo	Seed	Acetone	Goto-Kakizaki	250 mg/kg	1 h	Shinde et al. (2008)
In vivo	Seed	Ethyl acetate, ethanol	SID	200 mg/kg	15 d	Kumar et al. (2013)
In vivo	Seed	Mycaminose	SID	50 mg/kg	15 d	Kumar et al. (2013)
In vivo	Seed	Ethanol	AID	75 mg/100 g	15 d	Singh and Gupta (2007)
In vivo	Seed	Methanol	SID	100 mg/kg	21 d	Farswan et al. (2009)
In vivo	Seed	Cuminoside	SID	50 mg/kg	NS	Farswan et al. (2009)
In vivo	Seed	Aqueous	HFD and SID	200 mg/kg	21 d	Sharma et al. (2017)
In vivo	Stem	Aqueous	SID	4ml/kg	30 d	Swami et al. (2017)

Family, scientific name, level of evidence	Part used	Active extract / compound	Bioassay / model	Dose	Duration	Reference
**Nyctaginaceae**						
Boerhavia diffusa L.						
In vivo	Leaf	Aqueous	AID	200 mg/kg	4 week	Pari and Satheesh (2004)
**Oxalidaceae**						
Averrhoa carambola L.						
In vivo	Fruit	NA	SID	25 mg/kg	21 d	Pham et al. (2017)
In vivo	Leaf	Methanol	GLD	400 mg/kg	1 h	Shahreen et al. (2012)
In vivo	Leaf	Apigenin-6-C-(2"-O-α-rhamnopyranosyl)-β-fucopyranoside	NOR	20 mg/kg	180 min	Cazarolli et al. (2012)
In vivo	Leaf	Apigenin-6-C-β-fucopyranoside	NOR	20 mg/kg	180 min	Cazarolli et al. (2012)
In vivo	Root	2-dodecyl-6-methoxycycyclohexa-2,5-1,4-dione	HFD	12.5 mg/kg	16 week	Li et al. (2016)
**Plantaginaceae**						
Scoparia dulcis L.						
In vivo	Leaf	Aqueous	AID	0.45 g/kg	45 d	Pari and Venkateswaran (2002)
In vitro	NS	Methanol	AGI	80.35 μg/ml	NA	Mishra et al. (2013)
In vivo	NS	Methanol	SID	200 mg/kg	21 d	Mishra et al. (2013)
In vivo	NS	Aqueous	SID	50 mg/kg	3 week	Latha and Pari (2004)
In vivo	Whole plant	Scoparic acid D	SID	20 mg/kg	15 d	Latha et al. (2009)
In vivo	Whole plant	Aqueous	SID	200 mg/kg	15 d	Latha et al. (2004)

Family, scientific name, level of evidence	Part used	Active extract / compound	Bioassay / model	Dose	Duration	Reference
In vivo	Whole plant	Aqueous	SID	200 mg/kg	3 week	Pari and Latha (2005)
In vivo	Whole plant	Aqueous	SID	200 mg/kg	6 week	Latha and Pari, (2003); Pari and Latha, (2006)
**Poaceae**						
Eleusine coracana (L.) Gaertn.						
In vivo	Seed	Methanol	SID	NS	6 week	Shobana et al. (2010)
Clinical	NS	NA	T1D	NS	30 min	Urooj et al. (2006)
Setaria italica (L.) P.Beauv.						
In vitro	Seed	70% Ethanol	AGI	1.1 µg/ml	NA	Kim et al. (2011)
**Sapindaceae**						
Cardiospermum halicacabum L.						
In vivo	Leaf	Ethanol	SID	200 mg/ml	45 d	Veeramani et al. (2008)
